# Effective equations governing an active poroelastic medium

**DOI:** 10.1098/rspa.2016.0755

**Published:** 2017-02-22

**Authors:** J. Collis, D. L. Brown, M. E. Hubbard, R. D. O’Dea

**Affiliations:** School of Mathematical Sciences, University of Nottingham, University Park, Nottingham NG7 2RD, UK

**Keywords:** multiscale asymptotics, fluid–structure interaction, poroelasticity, growing media

## Abstract

In this work, we consider the spatial homogenization of a coupled transport and fluid–structure interaction model, to the end of deriving a system of effective equations describing the flow, elastic deformation and transport in an active poroelastic medium. The ‘active’ nature of the material results from a morphoelastic response to a chemical stimulant, in which the growth time scale is strongly separated from other elastic time scales. The resulting effective model is broadly relevant to the study of biological tissue growth, geophysical flows (e.g. swelling in coals and clays) and a wide range of industrial applications (e.g. absorbant hygiene products). The key contribution of this work is the derivation of a system of homogenized partial differential equations describing macroscale growth, coupled to transport of solute, that explicitly incorporates details of the structure and dynamics of the microscopic system, and, moreover, admits finite growth and deformation at the pore scale. The resulting macroscale model comprises a Biot-type system, augmented with additional terms pertaining to growth, coupled to an advection–reaction–diffusion equation. The resultant system of effective equations is then compared with other recent models under a selection of appropriate simplifying asymptotic limits.

## 1. Introduction

Poroelasticity is concerned with the study of elastic bodies that contain pore structures saturated with fluids. The characterization of poroelastic media has garnered much attention over the last 50 years across a wide range of fields studied by applied mathematicians and engineers. Of particular current importance is the study of poroelasticity in biological materials (e.g. in modelling solid tumours [1,2] or tissue engineering applications [3]) and the subsurface (e.g. in oil reservoir engineering, radioactive waste disposal, CO_2_ sequestration, hydraulic and thermal fracturing, and cavity generation [4,5]). While there are well-known equations governing poroelasticity at the so-called macroscopic lengthscale (i.e. a lengthscale much greater than that of the pores) [6–9], these laws typically require *ab initio* a statement of the constitutive laws describing the bulk properties of the solid and fluid components that are averaged volumetrically, irrespective of any underlying structure. As a result, any effective coefficients are meaningful only at the macroscopic scale and models must be parametrized via macroscopic experiments. Given these deficiencies, a model that explicitly accounts for pore-scale physics provides numerous benefits. In general, however, the underlying fluid–structure interaction (FSI) problems are highly complex, multiphysical and nonlinear coupled processes, for which direct simulation on complicated pore structures over multiple lengthscales is practically impossible. As such, effective models that explicitly incorporate pore-scale physics into a macroscopic model provide theoretical and computational benefits at the expense of a mathematically challenging homogenization process. It is beyond the scope of this work to present a comprehensive review and comparison of upscaling techniques that may be employed in the field of poroelasticity. However, in addition to multiscale homogenization, we wish to highlight other applicable techniques such as effective medium theory [10,11], mixture theory [12–14] and volume averaging [15,16]. For a more complete discussion we refer the reader to review articles that discuss upscaling in the wider fields of poroelasticity [17], flow in porous media [18,19] and solute transport [20].

In addition to the classical difficulties associated with poroelastic media, in many applications the solid is ‘active’; that is to say, not only does the solid undergo elastic deformation, but it is also growing/swelling (or equivalently shrinking)^1^ as a result of some physical, chemical or biological process. For example, in the context of biological tissue growth, we may view the biological material as a poroelastic medium that is subject to a nutrient-regulated growth law, whereby the mass and volume of the solid material increases over time. For sufficiently large growth rates, this will inevitably affect the macroscopic flow and passive transport of nutrient through the tissue. Similar effects are present in geophysical applications such as swelling in porous clays and coal [21], as well as in industrial media such as absorbent hygiene products, where electrochemical processes dominate [21–23]. In this work, we present a general formulation by which a range of such biologically or industrially motivated problems may be studied. In particular, we consider the derivation of a system of effective macroscopic equations governing a growing poroelastic medium together with passive transport of a solute which acts to regulate the growth dynamics of the medium, by means of two-scale asymptotics. While the techniques employed here may apply naturally to other formulations, here we forgo consideration of other forms of ‘active’ media, such as those that are thermo- or electromechanically active. Moreover, the growth law considered in the current work does not incorporate complex phase transition effects that would provide a more complete description of the underlying systems in the aforementioned applications.

Multiple-scale asymptotics allows the derivation of effective models at the macroscale that explicitly incorporate microstructural information. The application of these techniques is, however, meaningful only for problems in which there are multiple lengthscales that are well separated and there is sufficient uniformity (in the sense of periodicity) in the microscopic structure; see, for example, [24]. In this framework, local problems are derived that relate the microscopic and macroscopic structures, which may subsequently be employed in the construction of effective coefficients. The resultant models can be made rigorous via two-scale convergence, oscillatory test functions, etc. [25,26]. Though this is beyond the scope of this work, examples employing such methods include computational frameworks such as the multiscale finite element method [27,28] where the corrector estimates are utilized in error estimation. A wide range of biological applications employing multiscale methods may be found in the literature, including [29–34], for example.

Of particular relevance to this study are [35–37]. In [35], the authors present a rigorous derivation of the Biot model of poroelasticity [6,7] via multiple-scales expansions. In a recent work [36], an extension of the analysis in [35] is performed to consider a growing, elastic solid. The multiscale model in [36] permits finite microscale growth via an accretion growth law, though it makes the assumption of infinitesimal elastic deformation at the pore scale. Other recent works that consider the multiscale analysis of growing materials are also highly pertinent; see, for example, [38–40]. In [37], the authors consider the homogenization of an FSI system under finite pore-scale deformation in a common reference frame. Such an approach has also been applied successfully in homogenizing domains with evolving microstructure [41–43].

The study of growing material is of great importance in the biological sciences [44,45], and is a field in which there remain many open mathematical questions. One of the earliest applications of continuum mechanics in the study of growth of deformable biological materials was described in [46]. Later significant studies in the field include [47,48], which study both volumetric growth and accretion. However, the key reference in morphoelasticity (i.e. the study of growth in deformable media) is [49], in which a general formulation for finite volumetric growth in elastic tissues is proposed. Alternative proposals for growth models may also be found in [50,51]. While much of the literature pertaining to growth in deformable media is biologically focused, there are many applications in the physical sciences in which solid materials undergo volumetric changes as a result of external drivers such as temperature or the presence of chemical species. In particular, we highlight the similarity between biological growth, swelling in geological media such as clays and shales, and absorbent thin porous media in industrial applications described earlier.

The analysis we present in this work represents a significant extension of classical homogenization techniques of flow and transport in porous media. Here, we extend both the extensive literature pertaining to the homogenization of flow and transport in standard (i.e. not growing) porous media across the physical and biological sciences (see, for example [26,35,52,53]); and the recent attempts to apply these ideas to growing material in [36,38,39]. These studies typically place asymptotic restrictions on the underlying model to reduce the degree of nonlinearity (e.g. the linear coupling between fluid and solid mechanics employed in [36]) and/or enforce quasi-static conditions (e.g. the movement of the free interface as described in [38,39]). In this article, we present a framework in which we consider the fully coupled, nonlinear system of equations describing growth, transport and mechanics that results from finite growth and deformation at the pore scale when employing a growth model which neglects effects associated with complex phase transitions. The subsequent application of two-scale asymptotic techniques to this system of equations is further complicated by the fact that the equations governing the fluid and solid mechanics are most naturally written in different reference frames. As such, the system of equations describing the FSI does not yield a coherent understanding of the relationship between microscopic and macroscopic quantities because the periodicity assumption no longer holds. We proceed following the techniques set out in [37], whereby the FSI problem is written in a unified periodic domain, to which we apply asymptotic techniques. However, this work further represents a significant extension of that presented in [37], as here we both consider a hyperelastic solid material and employ a morphoelastic growth law along similar lines to that described in [49,54], as opposed to the linearly elastic inactive solid material considered therein.

This article is organized as follows. In §2, we introduce the fine-scale FSI model for the growing deformable medium. In §3, we rewrite all equations in a common periodic reference geometry, derive a system of cell equations at the microscale and effective macroscale equations, and summarize our new formulation. Then, in §4 we demonstrate the relationship between our new model and other recent models by considering appropriate limiting cases. Finally, in §5 we make concluding remarks and highlight ongoing and future work.

## 2. Model description

In this section, we first introduce the notation for the idealized representation of a poroelastic medium at the microscale in an initial reference configuration. We then discuss the general form of the growth law we consider, following closely that presented in [49,54], after which we present the equations governing the fluid motion, elastic deformation and solute transport. For the sake of generality and clarity of presentation, the model presented here is intentionally generic. Biological or physical motivation is therefore minimal, except where necessary for rationalizing specific modelling choices. Given this generality, the analysis and resultant effective equations presented here may prove applicable in many fields of study, though our primary motivations are biological tissue growth and hydrogeology.

Throughout this work, we denote by ∇_***ξ***_, ∇_***ξ***_⋅ and Δ_***ξ***_ the gradient, divergence and Laplacian, respectively, for differentiation with respect to the coordinate ***ξ***. For a vector field ***Υ***, rank 2 tensor fields ***A*** and ***B***, rank 3 tensor field A, and rank 4 tensor field 𝒜, we define

2.1 $${\left(\nabla_{\xi }\Upsilon \right)}_{ij}=\frac{\partial \Upsilon_{i}}{\partial \xi_{j}},\;{\left(\nabla_{\xi }A\right)}_{ijk}=\frac{\partial A_{ij}}{\partial \xi_{k}},\;{\left(\nabla_{\xi }A\right)}_{ijkl}=\frac{\partial A_{ijk}}{\partial \xi_{l}}\;\text{and}\;{\left(\nabla_{\xi }\cdot A\right)}_{i}=\frac{\partial A_{ij}}{\partial \xi_{j}},$$

and the contractions

2.2 $$A:B=A_{ij}B_{ij},\;{\left(A:B\right)}_{i}=A_{ijk}B_{jk}\;\text{and}\;{\left(𝒜:B\right)}_{ij}={𝒜}_{ijkl}B_{kl},$$

where we employ the Einstein summation convention over repeated indices. Finally, for a scalar function *ψ*(***A***), we define

2.3 $${\left(\frac{\partial \psi (A)}{\partial A}\right)}_{ij}=\frac{\partial \psi (A)}{\partial A_{ij}}.$$

Given the large amount of mathematical notation employed in the remainder of this article, we have included a brief summary of the nomenclature in table 1, given in appendix A.

**Table 1.** Nomenclature employed in the article.

nomenclature	description
*L*	lengthscale associated with macroscopic domain
ℓ	lengthscale associated with periodic microstructure
*ε*	ratio of microscopic and macroscopic lengthscales
$\tau_{l},\tau_{g},\tau_{D}$	time scales associated with loading, growth and diffusion
$\Omega_{\varepsilon }$	reference domain
${\overset{˚}{\Omega }}_{\varepsilon }(t)$	grown domain
${\tilde{\Omega }}_{\varepsilon }(t)$	elastically deformed domain
${\left(\cdot \right)}^{S}$	quantity related to solid domain
${\left(\cdot \right)}^{F}$	quantity related to fluid domain
$\Gamma_{\varepsilon }$	interface between solid and fluid domains
*ϕ*	porosity
$Y^{}$	periodic cell
$Y^{\Gamma }$	cell fluid–solid interface
***n***	normal on the fluid–solid interface
***τ***	tangents on the fluid–solid interface
$\overset{˚}{\chi }$	growth deformation
$\tilde{\chi }$	elastic deformation
°	variable defined on grown domain
˜	variable defined on elastically deformed domain
***F***	deformation gradient
***G***	Piola transformation
*J*	Jacobian
(·)_e_	transformation quantity associated with $\tilde{\chi }$
(·)_g_	transformation quantity associated with $\overset{˚}{\chi }$
*p*, ***v***	fluid pressure and velocity
$C_{e},\Psi$	Cauchy–Green tensor, strain energy functional
${\overset{˚}{\sigma }}_{\varepsilon }^{S}$	Piola stress in grown domain
***T***_e_	Piola stress in reference geometry
*c*, *D*, $R_{S}$	solute concentration, diffusivity and consumption
***f***	forcing
***x***	macroscale coordinate (slow moving)
***y***	microscale coordinate (fast moving)
(·)^(*i*)^	*i*th term in two-scale expansion
C	fourth-order stiffness tensor
E	symmetric strain tensor
***u***	displacement in the linearly elastic case

### (a) Idealized porous medium in the reference configuration

We consider an idealized porous medium in ℝ^*d*^, *d*=2,3. We model the medium as a highly connected material (i.e. both fluid and solid portions of the material are connected) with a (locally) spatially periodic microstructure comprising a growing, hyperelastic solid saturated with a viscous Newtonian fluid. Further, we consider the growth dynamics of the solid to be governed by the availability of a passive solute transported through the domain. We make the assumption that the porous material may be characterized by two distinct lengthscales: the lengthscale corresponding to the full extent of the material, denoted *L* and referred to as the macroscale, and that corresponding to the periodic microstructure, denoted ℓ and herein referred to as the microscale or pore scale. We assume that there is a strong separation of lengthscales; that is, the dimensionless parameter *ε*=ℓ/*L* satisfies 0<*ε*≪1. For simplicity in what follows, we shall scale the macroscopic parameter with unity, i.e. *L*=1, and *ε*=ℓ≪1.

We denote by *Ω*_*ε*_ the initial macroscale reference domain, which comprises the periodic microstructure, and denote the homogenized macroscopic domain by *Ω*. Throughout this work, we employ the subscript *ε* to signify dependence on the material’s microstructure. We further partition *Ω*_*ε*_ into two macroscopic subdomains $\Omega_{\varepsilon }^{F}$ and $\Omega_{\varepsilon }^{S}$ such that ${\bar{\Omega }}_{\varepsilon }^{F}\cup {\bar{\Omega }}_{\varepsilon }^{S}={\bar{\Omega }}_{\varepsilon }$ and $\Omega_{\varepsilon }^{F}\cap \Omega_{\varepsilon }^{S}=\emptyset$; where $\Omega_{\varepsilon }^{F}$ and $\Omega_{\varepsilon }^{S}$ correspond to the regions of *Ω*_*ε*_ containing fluid and solid material, respectively, and the notation ^-^$\bar{}$ denotes the closure of a domain. In addition, we assume that the fluid domain is sufficiently connected to obtain a non-trivial flow and the solid domain is sufficiently connected to prevent pieces of solid being carried away by the fluid. Furthermore, we denote by *Γ*_*ε*_ the initial reference interface between $\Omega_{\varepsilon }^{F}$ and $\Omega_{\varepsilon }^{S}$. Finally, we denote the unit inward normal to $\Omega_{\varepsilon }^{S}$ on *Γ*_*ε*_ by ***n***_*ε*_, and the unit tangent(s) to *Γ*_*ε*_ by ***τ***_*ε*_.

We specify that the microstructure may be characterized such that the fluid and solid subdomains may be decomposed into a set of unit cells ${\left\{Y_{i}^{S}\right\}}_{i\in I}$ and ${\left\{Y_{i}^{F}\right\}}_{i\in I}$, respectively, for some suitable index set I. Given the periodicity of the microstructure, each cell corresponds to a translation of a single reference cell; that is,

2.4 $$Y_{i}^{S}=Y^{S}+k_{i}\;\text{and}\;Y_{i}^{F}=Y^{F}+k_{i},\;\text{where }k_{i}\in Z^{d}\;\forall i\in I.$$

Under this notation we may decompose the solid and fluid domains as

2.5 $$\Omega_{\varepsilon }^{S}=\underset{k_{i}:i\in I}{⋃}\varepsilon (Y^{S}+k_{i})\cap \Omega_{\varepsilon }\;\text{and}\;\Omega_{\varepsilon }^{F}=\underset{k_{i}:i\in I}{⋃}\varepsilon (Y^{F}+k_{i})\cap \Omega_{\varepsilon }.$$

**Figure 1.**
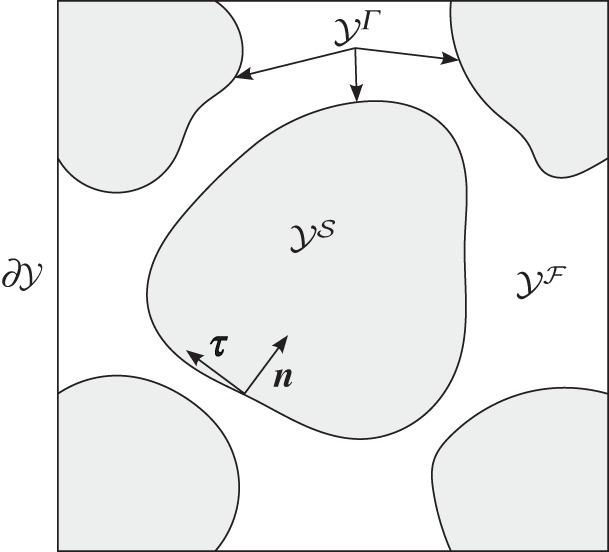
Schematic of the reference cell, $Y^{}$, decomposed into the fluid domain $Y^{F}$, the solid domain $Y^{S}$ and the interface $Y^{\Gamma }$. (Adapted from [39], fig. 1.)

We may further denote the reference cell by $Y^{}=Y^{S}\cup Y^{F}$ and denote the fluid–solid interface in the reference cell by $Y^{\Gamma }={\bar{Y}}^{S}\cap {\bar{Y}}^{F}$. Moreover, we denote the unit inward normal to $Y^{S}$ on $Y^{\Gamma }$ by ***n*** and tangents on $Y^{\Gamma }$ by ***τ***. A schematic diagram of the reference cell $Y^{}$ is shown in figure 1.

### (b) Morphoelastic growth law

We consider finite growth and deformation of the elastic body $\Omega_{\varepsilon }^{S}$, employing the theory of solid mechanics to describe the deformations of the body under the load and stress induced through the growth of the body and its interaction with the surrounding fluid. Following the decomposition first described in [49] in the field of morphoelasticity, given an initial, residual stress-free reference configuration the model of growth employed here may be described in two stages:

(i) We consider a geometric (stress-free) deformation of the body which characterizes the physical/chemical/biological processes governing growth to obtain a *virtual grown configuration*.
(ii) We then consider the elastic response of this grown body as a means of enforcing physical compatibility and the physical constraints imposed on the elastic body via its interactions with the surrounding fluid and geometry to obtain the *current deformed configuration*.

This second stage is of crucial importance as we make the assumption that the growth process itself is entirely local at each point, that is, independent of the growth nearby. Given such an assumption, it is possible that non-physical configurations may arise as a result of the initial growth stage and, as such, the elastic response is necessary to obtain physically meaningful results.

**Figure 2.**
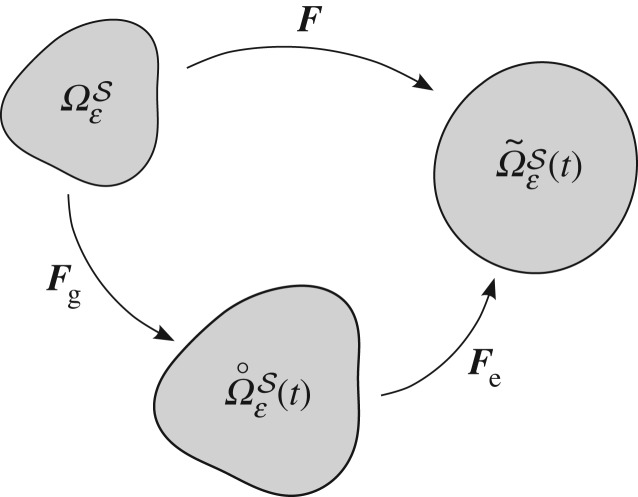
Schematic diagram of the decomposition into the growth and elastic response deformations. Where ***F***, ***F***_g_ and ***F***_e_ denote the total, growth and elastic deformations and $\Omega_{\varepsilon }$, $\overset{˚}{\Omega_{\varepsilon }}(t)$ and $\tilde{\Omega_{\varepsilon }}(t)$ denote the initial reference, virtual grown and current deformed configurations, respectively.

Extending the notation introduced in §2a, we denote the virtual grown configuration by ${\overset{˚}{\Omega }}_{\varepsilon }^{S}$ and the current deformed configuration by ${\tilde{\Omega }}_{\varepsilon }^{S}$. We now specify that the deformation $\Omega_{\varepsilon }^{S}\to {\tilde{\Omega }}_{\varepsilon }^{S}$ may be characterized by the deformation gradient ***F***. The key tenet of morphoelasticity, introduced in [49], is that ***F*** may be decomposed into the composition of a tensor describing growth, denoted ***F***_g_, and an elastic deformation, denoted ***F***_e_, i.e. ***F***=***F***_e_°***F***_g_. A schematic diagram demonstrating the decomposition of the deformation and the notation for the variously transformed domains is given in figure 2. This approach has been adopted widely in the field of biomedical engineering and we refer to the review articles [55,56] for discussion regarding its application. We highlight, for example, the application to cardiac [57], arterial [45] and skin [58] tissue growth models, and the comparisons against relevant clinical/experimental data therein, in particular.

While, conceptually, this decomposition appears natural, there is a subtlety in its application to time-dependent continuous growth, which we discuss briefly, following closely the exposition given in [54]. In growth mechanics, there are typically four time scales of interest: corresponding to elastic wave propagation (*τ*_e_), viscoelastic relaxation (*τ*_*v*_), external loading (*τ*_*l*_) and growth (*τ*_g_). Implicit in the above is the assumption that the decomposition is instantaneous, insomuch as it applies continuously in time and, as ***F***_g_ evolves, ***F***_e_ responds instantaneously. This requires strong separation between the growth time scale and the elastic time scales. In the following analysis, we neglect elastic wave propogation and viscoelastic effects and, as such, make no further reference to *τ*_e_ and *τ*_v_,^2^ and concentrate solely on effects occurring on time scales *τ*_l_ and *τ*_g_ in the remainder of this article. While there may be regimes in which growth time scales become comparable to others, we assume here that growth time scales are larger than the other pertinent time scale with

2.6 $$\tau_{l}\ll \tau_{g},$$

as in [54]. Under this ordering of the time scales, elastic responses of the material occur much quicker than growth, so that for time smaller than *τ*_g_ the solid material is in a quasi-static elastic equilibrium. As such, we consider that the only pertinent time variation is that associated with the evolution of the growth tensor given by

2.7 $$\frac{dF_{g}}{dt}=ℋ(F_{e},F_{g},\ldots ;t).$$

We now consider a time increment *δt*, such that *τ*_l_≪*δt*≪*τ*_g_, and apply a single time step of a forward Euler method to (2.7) to obtain

2.8 $$F_{g}(t+\delta t)=F_{g}(t)+\delta tℋ(F_{e},F_{g},\ldots ;t).$$

This permits the definition of an incremental growth and elastic deformation associated with *δt* by

**Figure 3.**
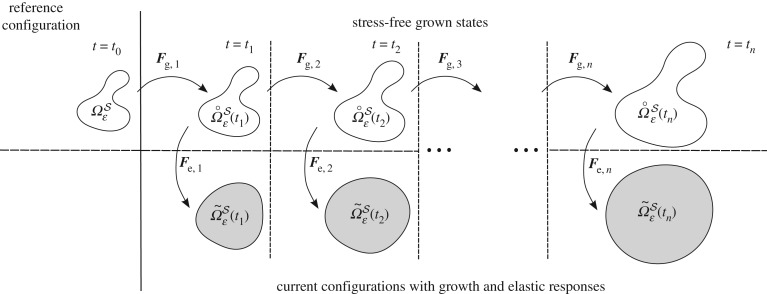
Schematic diagram of the decomposition into the growth and elastic response deformations for multiple incremental growth steps, where ***F***_g,*i*_ and ***F***_e,*i*_ denote the growth and elastic deformations to $\overset{˚}{\Omega_{\varepsilon }}(t_{i})$ and $\tilde{\Omega_{\varepsilon }}(t_{i})$, respectively, associated with transition from time *t*_*i*−1_ to *t*_*i*_.

2.9 $$F_{}^{\mathrm{inc}}=F_{e}^{\mathrm{inc}}\circ F_{g}^{\mathrm{inc}},$$

where $F_{g}^{\mathrm{inc}}:=\delta t$*ℋ*. In the following, we consider only incremental growth deformation gradients and as such forgo the notation associated with incremental growth for presentational convenience. The above definition of incremental growth and response provides a natural means of considering many growth steps. A schematic diagram demonstrating the application of multiple growth steps is given in figure 3. In the multiple-scales analysis presented in §3, we consider a single time increment only for the sake of concision, though we remark that the homogenization process generalizes naturally to any number of time steps.

As a means of modelling either nutrient-regulated growth in biological applications or chemical-regulated degradation in industrial applications, we additionally specify that the growth is coupled to transport. Under this assumption, ***F***_g_ has a functional dependence on the concentration of solute, denoted *c*_*ε*_. For simplicity, we set ***F***_g_=***F***_g_(*c*_*ε*_) and do not consider explicit stress or time dependencies on growth. Additionally, we consider the transport of solute on the diffusive time scale *τ*_D_. Whether there is a strong separation between *τ*_g_ and *τ*_D_ is application specific and, therefore, in the following analysis we make no assumptions regarding the separation between these two scales. Further restrictions on the constitutive assumption for the underlying growth law are described in §2f.

In order for us to specify correctly the equations governing fluid and solid mechanics, and transport of the passive solute, we must define appropriate coordinate systems in the reference, virtual grown and deformed current configurations. As such, consider the mappings

2.10 $${\overset{˚}{\chi }}_{\varepsilon }(t):\Omega_{\varepsilon }\to {\overset{˚}{\Omega }}_{\varepsilon }(t)\;\text{and}\;{\tilde{\chi }}_{\varepsilon }(t):{\overset{˚}{\Omega }}_{\varepsilon }(t)\to {\tilde{\Omega }}_{\varepsilon }(t),$$

whereby ${\overset{˚}{\chi }}_{\varepsilon }$ is obtained by appropriate constitutive assumptions on the growth dynamics, and ${\tilde{\chi }}_{\varepsilon }$ is obtained via solution of an elasticity problem. Under these definitions, a point *P* in $\Omega_{\varepsilon }$ with coordinates ***x*** at time *t* has coordinates $\overset{˚}{x}={\overset{˚}{\chi }}_{\varepsilon }(x,t)$ in ${\overset{˚}{\Omega }}_{\varepsilon }(t)$ and $\tilde{x}={\tilde{\chi }}_{\varepsilon }(\overset{˚}{x},t)$ in ${\tilde{\Omega }}_{\varepsilon }(t)$. Moreover, we may naturally define the grown and deformed full domains, fluid domains and interface by

$$\begin{array}{rl} {\overset{˚}{\Omega }}_{\varepsilon }(t) & =\{{\overset{˚}{\chi }}_{\varepsilon }(x,t):x\in \Omega_{\varepsilon }\},\;{\tilde{\Omega }}_{\varepsilon }(t)=\{{\tilde{\chi }}_{\varepsilon }\circ {\overset{˚}{\chi }}_{\varepsilon }(x,t):x\in \Omega_{\varepsilon }\},\\ {\overset{˚}{\Omega }}_{\varepsilon }^{F}(t) & =\{{\overset{˚}{\chi }}_{\varepsilon }(x,t):x\in \Omega_{\varepsilon }^{F}\},\;{\tilde{\Omega }}_{\varepsilon }^{F}(t)=\{{\tilde{\chi }}_{\varepsilon }\circ {\overset{˚}{\chi }}_{\varepsilon }(x,t):x\in \Omega_{\varepsilon }^{F}\},\\ {\overset{˚}{\Gamma }}_{\varepsilon }(t) & =\{{\overset{˚}{\chi }}_{\varepsilon }(x,t):x\in \Gamma_{\varepsilon }\},\;{\tilde{\Gamma }}_{\varepsilon }(t)=\{{\tilde{\chi }}_{\varepsilon }\circ {\overset{˚}{\chi }}_{\varepsilon }(x,t):x\in \Gamma_{\varepsilon }\}. \end{array}$$

For clarity, in the following we further identify all dependent variables defined with respect to the virtual grown configuration or the current deformed configuration by ${\;}^{{\;}^{\overset{˚}{\;}}}$ or $\tilde{\;}$, respectively. Finally, we remark that we defer explicit definition of the deformations ${\overset{˚}{\chi }}_{\varepsilon }$ and ${\tilde{\chi }}_{\varepsilon }$ until the definition of the constitutive assumption on the equations governing the elastic deformation in §2d and the growth law given in §2f.

### (c) Fluid equations

The motion of the fluid is governed by the incompressible Navier–Stokes equations; however, following the arguments presented in [38,39,59] we assume that the microstructure and fluid velocity are scaled such that the time derivative and inertial terms in the Navier–Stokes equations are $O(\varepsilon^{2})$. Further, we note that these equations are most naturally presented in the current deformed configuration. Denoting the pressure, velocity and dynamic viscosity of the fluid by ${\tilde{p}}_{\varepsilon }$, ${\tilde{v}}_{\varepsilon }$ and *μ*, respectively, the fluid in ${\tilde{\Omega }}_{\varepsilon }^{F}(t)$ is governed by the Stokes equations

2.11 $$\begin{array}{rl} -\nabla_{\tilde{x}}{\tilde{p}}_{\varepsilon }+\mu \nabla_{\tilde{x}}\cdot (\nabla_{\tilde{x}}{\tilde{v}}_{\varepsilon }+{\left(\nabla_{\tilde{x}}{\tilde{v}}_{\varepsilon }\right)}^{T})={\tilde{f}}_{\varepsilon }^{F} & \;\forall \tilde{x}\in {\tilde{\Omega }}_{\varepsilon }^{F}(t) \end{array}$$

and

2.12 $$\begin{array}{rl} \nabla_{\tilde{x}}\cdot {\tilde{v}}_{\varepsilon }=0\; & \;\forall \tilde{x}\in {\tilde{\Omega }}_{\varepsilon }^{F}(t), \end{array}$$

where ${\tilde{f}}_{\varepsilon }^{F}$ denotes an external force acting on the fluid. We note that, while the momentum equation (2.11) is quasi-steady, ${\tilde{p}}_{\varepsilon }$ and ${\tilde{v}}_{\varepsilon }$ have implicit time dependence due to the growth and mechanics of the solid material.

It remains for us to specify the conditions governing the flow on the interface ${\tilde{\Gamma }}_{\varepsilon }$ and the remainder of the boundary $\partial {\tilde{\Omega }}_{\varepsilon }$. As this requires coupling with the solid equations (which are described in the grown domain) and periodicity (which is applicable only in the initial reference configuration), we defer their specification until §2g, once appropriate coordinate transformations have been defined.

### (d) Solid equations

We assume that the solid material is hyperelastic, i.e. the constitutive assumption on its stress may be determined via an appropriate strain energy functional. We proceed now by specifying the equations governing the deformation of the solid, presented in the current grown configuration (see [60,61]). Recalling the notation introduced in §2b, we define the elastic deformation gradient by

2.13 $$F_{e}:=\nabla_{\overset{˚}{x}}{\tilde{\chi }}_{\varepsilon },$$

and the right Cauchy–Green deformation tensor by

2.14 $$C_{e}:=F_{e}^{T}F_{e}.$$

In view of the time-scale separation (2.6), the solid skeleton satisfies

2.15 $$-\nabla_{\overset{˚}{x}}\cdot {\overset{˚}{\sigma }}_{\varepsilon }^{S}={\overset{˚}{f}}_{\varepsilon }^{S}\;\forall \overset{˚}{x}\in {\overset{˚}{\Omega }}_{\varepsilon }^{S},$$

where ${\overset{˚}{f}}_{\varepsilon }^{S}$ denotes a body force acting on the solid material and ${\overset{˚}{\sigma }}_{\varepsilon }^{S}$ denotes the Piola stress in the body. As we consider a hyperelastic material, we define ${\overset{˚}{\sigma }}_{\varepsilon }^{S}$ constitutively by

2.16 $${\overset{˚}{\sigma }}_{\varepsilon }^{S}:=2F_{e}\frac{\partial \Psi (C_{e})}{\partial C_{e}},$$

where $\Psi (C_{e})$ denotes the strain energy functional for a given material. In the analysis presented in §3b(ii), we require that the strain energy functional satisfies certain convexity requirements and, as such, we restrict our attention to materials whose strain energy functionals are strictly polyconvex (for a definition see, for example, [61]) such as Ogden [62] or Mooney–Rivlin [63,64] solids (commonly employed when modelling rubbers, polymers and biological tissues). Once more, we defer the specification of suitable interface and boundary conditions to §2g, after the definition of relevant coordinate transformation tensors.

### (e) Solute transport

In addition to considering the motion of the fluid and solid materials, we further model the transport of a passive solute whose concentration we denote $c_{\varepsilon }$. As we consider growth regulated by the passive solute, the evolutions of the fluid and solid domains are coupled to $c_{\varepsilon }$ as well as to each other through the FSI. In the fluid and solid domains (${\tilde{\Omega }}_{\varepsilon }^{F}$ and ${\tilde{\Omega }}_{\varepsilon }^{S}$, respectively), we assume that the solute is transported via advection and diffusion (in the latter, the advection arising due to the growth and deformation of the solid material). In the solid, however, we consider an additional consumption term associated with growth. Finally, we note that the equations governing the transport of this solute in each sub-domain are most naturally posed in the current deformed configuration as diffusive fluxes are associated with spatial concentration gradients, as opposed to material gradients in the grown or initial configurations.

Given these assumptions, the equations governing the evolution of this transported species in ${\tilde{\Omega }}_{\varepsilon }^{F}$ is given by

2.17 $$\frac{\partial {\tilde{c}}_{\varepsilon }}{\partial t}+\nabla_{\tilde{x}}\cdot ({\tilde{v}}_{\varepsilon }{\tilde{c}}_{\varepsilon })=D_{F}\Delta_{\tilde{x}}{\tilde{c}}_{\varepsilon }\;\forall \tilde{x}\in {\tilde{\Omega }}_{\varepsilon }^{F},$$

where $D_{F}$ denotes the diffusivity of the solute in the fluid; and the evolution in ${\tilde{\Omega }}_{\varepsilon }^{S}$ is given by

2.18 $$\frac{\partial {\tilde{c}}_{\varepsilon }}{\partial t}+\nabla_{\tilde{x}}\cdot \left(\frac{\partial \tilde{\chi }}{\partial t}{\tilde{c}}_{\varepsilon }\right)=D_{S}\Delta_{\tilde{x}}{\tilde{c}}_{\varepsilon }-R_{S}{\tilde{c}}_{\varepsilon }\;\forall \tilde{x}\in {\tilde{\Omega }}_{\varepsilon }^{S},$$

where $D_{S}$ denotes the diffusivity of the solute and $R_{S}$ is a constant that denotes the consumption of solute in the solid material. We note that in general $R_{S}$ may have a complicated dependence on a range of model variables which we neglect here for the sake of clarity of presentation.

### (f) Assumption on the growth law

We now specify assumptions on growth that we employ in the following analysis. For the sake of simplicity, we consider that the growth of the solid material is isotropic and a function of the local concentration of solute only. Under these assumptions, the natural form of the growth displacement is given by

2.19 $${\overset{˚}{\chi }}_{\varepsilon }(x)={\overset{˚}{\chi }}_{\varepsilon }(x,c_{\varepsilon }),$$

in which there is an implicit time dependence provided by the coupling to $c_{\varepsilon }(x,t)$. The deformation gradient associated with the growth deformation is then given by

2.20 $$F_{g}:=\nabla_{x}{\overset{˚}{\chi }}_{\varepsilon }(x,c_{\varepsilon }).$$

We note that for simplicity, and as we wish to maintain a general formulation in this study, the growth law employed here does not incorporate complicated phase transitions and stress dependence that are often employed in the modelling of biological materials. We refer the reader to, for example, [56] for further discussion on growth laws in a biological setting. In particular, we highlight the lack of non-local terms corresponding to consumption of the fluid phase that may occur in certain applications described previously under the assumption of a more complex growth law.

### (g) Interface conditions

In this section, we specify the interface conditions for the fluid–structure coupling, and the transport of solute. Firstly, we introduce notation for the jump of a quantity across the interface. Recalling the definition of $n_{\varepsilon }$, we define the trace of a scalar quantity *ψ* (defined in $\Omega_{\varepsilon }^{F}$ and $\Omega_{\varepsilon }^{S}$), on $\Gamma_{\varepsilon }$ by

2.21 $$\psi^{\pm }:=\lim_{\epsilon \to 0}\psi (x\pm \epsilon n_{\varepsilon }).$$

Under this notation, we define the jump operator by

2.22 $$[[\psi ]]=\psi^{+}-\psi^{-},$$

where we extend to vector and tensor quantities componentwise according to this definition.

#### (i) Fluid–structure coupling

The natural conditions to impose in the current context of FSI are continuity of velocity and continuity of total stress. However, a complication lies in the fact that the fluid and solid equations are presented with respect to different configurations (i.e. ${\overset{˚}{\Omega }}_{\varepsilon }(t)$ and ${\tilde{\Omega }}_{\varepsilon }(t)$, respectively). We must therefore consider appropriate transformations of the fluid stress, in view of which we defer the definition of the interface conditions to §3a, wherein we describe the process of mapping the FSI problem to a unified periodic domain.

#### (ii) Solute

Suitable conditions for the concentration of the passive solute are continuity of solute concentration and flux across the interface, i.e.

2.23 $$[[D\nabla_{\tilde{x}}{\tilde{c}}_{\varepsilon }\cdot {\tilde{n}}_{\varepsilon }(t)]]=0\;\forall \tilde{x}\in {\tilde{\Gamma }}_{\varepsilon }$$

and

2.24 $$[[{\tilde{c}}_{\varepsilon }]]=0\;\;\;\forall \tilde{x}\in {\tilde{\Gamma }}_{\varepsilon }.$$

As in the case of the fluid–structure coupling, we will subsequently be required to map these conditions onto the interface in the initial reference configuration when we come to perform the multiple-scale asymptotic analysis.

## 3. Homogenization in the Lagrangian frame

In this section, we describe the process of mapping the system of equations describing the FSI in §2c,d, and the solute transport in §2e, to a periodic reference geometry on which we may perform the two-scale asymptotic analysis. We then proceed by applying two-scale expansions and spatially averaging to obtain cell problems on the microscale (i.e. problems posed on the periodic cell $Y^{}$), and effective equations in the homogenized macroscale domain *Ω*.

### (a) Coordinate transformations

In this section, we apply a coordinate transformation to the equations governing the FSI and solute transport, together with the appropriate interface conditions, to yield a full system of equations on the fixed reference configuration denoted $\Omega_{\varepsilon }$. This process is analogous to the so-called *arbitrary Lagrangian–Eulerian* (ALE) formulation widely employed in computational studies; see [37,65].

Recalling from §2d,f the definitions of ***F***_e_ and ***F***_g_, we observe that these tensors are well defined only in ${\overset{˚}{\Omega }}_{\varepsilon }^{S}$. As we require both of these quantities to be defined throughout ${\overset{˚}{\Omega }}_{\varepsilon }$, we extend their definition to all of ${\overset{˚}{\Omega }}_{\varepsilon }$ by means of a suitable harmonic extension. We now define the Piola transformations, ***G***_*α*_, and Jacobians, *J*_*α*_, by

3.1 $$J_{\alpha }=\det F_{\alpha }\;\text{and}\;G_{\alpha }=J_{\alpha }F_{\alpha }^{-1}\;\text{for }\alpha \in \{e,g\}.$$

Following [65], we are able to obtain the identities for the transformation of derivatives under a generic mapping. Consider a general mapping ${\overset{ˇ}{\chi }}_{\varepsilon }$ defined by

3.2 $$\begin{array}{rl} {\overset{ˇ}{\chi }}_{\varepsilon }:\; & {\hat{\Omega }}_{\varepsilon }\to {\overset{ˇ}{\Omega }}_{\varepsilon }\\ \;\text{and}\; & \;\hat{x}\mapsto \overset{ˇ}{x}. \end{array}\}$$

We denote the associated gradient of the mapping by $F_{\#}=\nabla_{\hat{x}}{\overset{ˇ}{\chi }}_{\varepsilon }$, and define ***G***_#_ and *J*_#_ according to (3.1). Then, denoting a generic scalar field *ξ*, vector field Υ and tensor field **A**, and adopting the convention of using $\hat{\;}$ and $\overset{ˇ}{\;}$ to denote evaluation in ${\hat{\Omega }}_{\varepsilon }$ and ${\overset{ˇ}{\Omega }}_{\varepsilon }$, respectively, application of the chain rule yields

3.3 $$\nabla_{\overset{ˇ}{x}}\overset{ˇ}{\xi }=F_{\#}^{-T}\nabla_{\hat{x}}\hat{\xi }\;\text{and}\;\nabla_{\overset{ˇ}{x}}\overset{ˇ}{\Upsilon }=(\nabla_{\hat{x}}\hat{\Upsilon })F_{\#}^{-1}.$$

Via the application of Nanson’s formula and the divergence theorem, we obtain

3.4 $$\nabla_{\overset{ˇ}{x}}\cdot \overset{ˇ}{\Upsilon }=\frac{1}{J_{\#}}\nabla_{\hat{x}}\cdot (J_{\#}F_{\#}^{-1}\hat{\Upsilon })\;\text{and}\;\nabla_{\overset{ˇ}{x}}\cdot \overset{ˇ}{\text{A}}=\frac{1}{J_{\#}}\nabla_{\hat{x}}\cdot (J_{\#}\hat{\text{A}}F_{\#}^{-T}).$$

Lastly, the chain rule provides

3.5 $$\frac{\partial \overset{ˇ}{\xi }}{\partial t}=\frac{\partial \hat{\xi }}{\partial t}-\left(F_{\#}^{-1}\frac{\partial {\overset{ˇ}{\chi }}_{\varepsilon }}{\partial t}\cdot \nabla_{\hat{x}}\right)\hat{\xi }.$$

By substituting the appropriate nomenclature associated with the mappings ${\overset{˚}{\chi }}_{\varepsilon }$ and ${\tilde{\chi }}_{\varepsilon }$ into (3.3)–(3.5), we are able to obtain expressions for mapping derivatives between the reference, virtual grown and current deformed configurations.

We now proceed to write the coupled FSI, growth and transport problem in the initial reference configuration employing (3.3)–(3.5). The first stage of this process is to rewrite these equations in the virtual grown configuration, thus obtaining a system equivalent to that obtained in the ALE frame in [37]. The second stage, which differentiates this work from [37], is to further map the equations obtained in the grown configuration, ${\overset{˚}{\Omega }}_{\varepsilon }$, to the periodic reference configuration $\Omega_{\varepsilon }$. To this end, and for the sake of concision, we introduce the following notation corresponding to the combination of growth and elastic deformation:

3.6 $$F_{}:=F_{g}F_{e},\;G_{}:=G_{g}G_{e}\;\text{and}\;J_{}:=J_{g}J_{e}.$$

The equations governing the fluid are thus given by

3.7 $$\begin{array}{rl} -G_{}^{T}\nabla_{x}p_{\varepsilon }+\mu \nabla_{x}\cdot ((\nabla_{x}v_{\varepsilon })G_{}F_{}^{-T}+G_{}^{T}{\left(\nabla_{x}v_{\varepsilon }\right)}^{T}F_{}^{-T})=J_{}f_{\varepsilon }^{F} & \;\forall x\in \Omega_{\varepsilon }^{F} \end{array}$$

and

3.8 $$\begin{array}{rl} \nabla_{x}\cdot (G_{}v_{\varepsilon })=0\; & \;\forall x\in \Omega_{\varepsilon }^{F}. \end{array}$$

The equations governing the elastic deformation are given by

3.9 $$-\nabla_{x}\cdot (\sigma_{\varepsilon }^{S}G_{g}^{T})=J_{g}f_{\varepsilon }^{S}\;\forall x\in \Omega_{\varepsilon }^{S}.$$

The equations governing the transport in the fluid domain are

3.10 $$J_{}\left(\frac{\partial c_{\varepsilon }}{\partial t}-2\left(F_{g}^{-1}\frac{\partial {\overset{˚}{\chi }}_{\varepsilon }}{\partial t}\cdot \nabla_{x}\right)c_{\varepsilon }\right)+\nabla_{x}\cdot (c_{\varepsilon }G_{}v_{\varepsilon })=D_{F}\nabla_{x}\cdot (G_{}F_{}^{-T}\nabla_{x}c_{\varepsilon })\;\forall x\in \Omega_{\varepsilon }^{F},$$

and in the solid domain

3.11 $$\begin{array}{rl} & J_{}\left(\frac{\partial c_{\varepsilon }}{\partial t}-\left(F_{g}^{-1}\frac{\partial {\overset{˚}{\chi }}_{\varepsilon }}{\partial t}\cdot \nabla_{x}\right)c_{\varepsilon }\right)+\nabla_{x}\cdot \left(J_{}F_{g}^{-1}\frac{\partial {\overset{˚}{\chi }}_{\varepsilon }}{\partial t}\right)c_{\varepsilon }\\ & \;=D_{S}\nabla_{x}\cdot (G_{}F_{}^{-T}\nabla_{x}c_{\varepsilon })+J_{}R_{S}c_{\varepsilon }\;\forall x\in \Omega_{\varepsilon }^{S}. \end{array}$$

We note that, despite the elastic deformation affecting transport, the time derivative of the elastic deformation does not appear in (3.10) explicitly due to our choice of temporal scalings. The coupling of the fluid and solid problems is specified via the velocity condition

3.12 $$v_{\varepsilon }=g_{\varepsilon }\;\forall x\in \Gamma_{\varepsilon },$$

where $g_{\varepsilon }$ denotes the interfacial growth velocity defined by

3.13 $$g_{\varepsilon }:=F_{e}\frac{\partial {\overset{˚}{\chi }}_{\varepsilon }}{\partial t},$$

and the stress condition

3.14 $$\sigma_{\varepsilon }^{F}G_{}^{T}n_{\varepsilon }=\sigma_{\varepsilon }^{S}G_{g}^{T}n_{\varepsilon }\;\forall x\in \Gamma_{\varepsilon },$$

where $\sigma_{\varepsilon }^{F}$ is the fluid stress, defined by

3.15 $$\sigma_{\varepsilon }^{F}:=-p_{\varepsilon }I+\mu ((\nabla_{x}v_{\varepsilon })F_{}^{-1}+F_{}^{-T}{\left(\nabla_{x}v_{\varepsilon }\right)}^{T}).$$

Finally, the coupling between the concentration of solute is given by

3.16 $$[[c_{\varepsilon }]]=0\;\text{and}\;[[D\nabla_{x}c_{\varepsilon }\cdot n_{\varepsilon }]]=0\;\forall x\in \Gamma_{\varepsilon }.$$

### (b) Multiscale homogenization

We now analyse the coupled system describing the FSI, growth and transport of the passive solute in the fixed reference configuration derived in §3a, with the aim of obtaining a macroscopic description in a manner analogous to that presented in [35–37]. Given the structure of the medium introduced in §2, we naturally define

3.17 $$y:=\frac{1}{\varepsilon }x$$

to be the spatial coordinate associated with the microscale (or *fast moving* coordinate), where ***x*** now corresponds to the spatial coordinate associated with the macroscale (or *slow moving* coordinate). Under the assumption of strong separation of scales, we may expand each dependent variable *ψ* in multiple-scales form via an expansion

3.18 $$\psi (x,y,t;\varepsilon )=\sum_{i=0}^{\infty }\varepsilon^{i}\psi^{\left(i\right)}(x,y,t).$$

Moreover, under this coordinate transformation the gradient operator ∇_***x***_ is transformed as

3.19 $$\nabla_{x}\to \nabla_{x}+\frac{1}{\varepsilon }\nabla_{y},$$

where $\nabla_{x}$ and $\nabla_{y}$ denote differentiation with respect to the macroscale and microscale spatial variables, respectively. We proceed by writing expansions of the form (3.17) for the dependent variables $c_{\varepsilon },{\tilde{\chi }}_{\varepsilon }$ and $p_{\varepsilon }$. Following [24,35–37], we then assume a standard scaling for Stokes flow and expand $v_{\varepsilon }$ according to

3.20 $$v_{\varepsilon }(x,y,t;\varepsilon )=\varepsilon^{2}\sum_{i=0}^{\infty }\varepsilon^{i}v^{\left(i\right)}(x,y,t),$$

and in order to maintain the correct scaling we follow [37,59] and scale the interfacial growth velocity employed in (3.11) with

3.21 $$g_{\varepsilon }=\varepsilon^{2}g.$$

We emphasize that this scaling ensures that growth and flow are of equal importance, rather than a simplifying relegation to lower order as commonly employed elsewhere. In particular, it implies that, while growth is finite, it is slow, consistent with (3.19).

As in [37], we expand ***F***_e_, *J*_e_ and ***G***_e_ according to (3.17), and we further propose the expansion of ***F***_g_, *J*_g_ and ***G***_g_ in a similar manner. We note that in §3b(i) we are required to assume that the proposed expansions for ***F***_e_, *J*_e_ and ***G***_e_ hold, i.e. the leading-order terms are O(1). However, we subsequently show, in §3b(ii), that under the assumption of strict polyconvexity of the strain energy functional this assumption is indeed valid and independent of the analysis presented in §3b(i). We constitutively specify $F_{g}=F_{g}(c_{\varepsilon })$ and subsequently show (see §3b(iii)) that at leading order the solute concentration exhibits no microscale dependence (as is typical of similar models [38,39]). Correspondingly, we may reasonably assume that ${\overset{˚}{\chi }}_{\varepsilon }$ is sufficiently smooth that the growth deformation gradient may be expanded as

3.22 $$F_{g}=F_{g}^{\left(0\right)}(x)+\varepsilon F_{g}^{\left(1\right)}(x,y)+O(\varepsilon^{2}),$$

where such an assumption permits the following analysis. Following this, we may expand the Jacobian for the growth deformation as

3.23 $$\begin{array}{rl} J_{g} & =J_{g}^{\left(0\right)}(x)+\varepsilon J_{g}^{\left(1\right)}(x,y)+O(\varepsilon^{2})\\ & =\det(F_{g}^{\left(0\right)}(x))+\varepsilon J_{g}^{\left(1\right)}(x,y)+O(\varepsilon^{2}). \end{array}$$

We note that it is straightforward to obtain expressions for *J*^(1)^_g_ in terms of the components of ***F***_g_; however, a precise expression for this term is not required for the analysis that follows and so is omitted for concision. For a generic tensor ***A***, under the assumption that *ε* is sufficiently small that

$${∥-\sum_{i=1}^{\infty }\varepsilon^{i}A^{\left(i\right)}{\left(A^{\left(0\right)}\right)}^{-1}∥}_{\mathrm{op}}<1,$$

we may obtain, by application of a Neumann series, the following expansion for its inverse, ***A***^−1^,

3.24 $$\begin{array}{rl} A^{-1} & =\sum_{j=0}^{\infty }{\left(-\sum_{i=1}^{\infty }\varepsilon^{i}A^{\left(i\right)}{\left(A^{\left(0\right)}\right)}^{-1}\right)}^{j}{\left(A^{\left(0\right)}\right)}^{-1}\\ & ={\left(A^{\left(0\right)}\right)}^{-1}-\varepsilon A^{\left(1\right)}{\left(A^{\left(0\right)}\right)}^{-2}+\ldots \\ & =\sum_{i=0}\varepsilon^{i}{\left(A^{-1}\right)}^{\left(i\right)}. \end{array}$$

Given this observation, we note that we may write the Piola transformation associated with growth as

3.25 $$\begin{array}{rl} G_{g} & =G_{g}^{\left(0\right)}(x)+\varepsilon G_{g}^{\left(1\right)}(x,y)+O(\varepsilon^{2})\\ & =\det(F_{g}^{\left(0\right)}(x)){\left(F_{g}^{\left(0\right)}(x)\right)}^{-1}+\varepsilon G_{g}^{\left(1\right)}(x,y)+O(\varepsilon^{2}). \end{array}$$

As above, an explicit expression for ***G***^(1)^_g_ is straightforward to obtain but is not included.

**Remark 3.1**

While we consider here a growth law that is dependent on the concentration of a solute, the following analysis naturally generalizes to a growth law that is dependent on any quantity that is microscale invariant to leading order.

Finally, we substitute each of these expansions, together with (3.18), into the system of equations set out in §3a to obtain a system of PDEs at increasing orders of *ε*.

To obtain the homogenized macroscale equations in the following sections, we take spatial averages of leading-order behaviour. To this end, we define the following integral averages associated with the reference cell:

3.26 $${\left\langle \psi \right\rangle }_{Y^{F}}=\frac{1}{\left|Y^{}\right|}\int_{Y^{F}}\psi \;dy,\;{\left\langle \psi \right\rangle }_{Y^{S}}=\frac{1}{\left|Y^{}\right|}\int_{Y^{S}}\psi \;dy\;\text{and}\;{\left\langle \psi \right\rangle }_{Y^{\Gamma }}=\frac{1}{\left|Y^{}\right|}\int_{Y^{\Gamma }}\psi \;ds,$$

and the material porosity, *ϕ*, by

3.27 $$\phi =\frac{\left|Y^{F}\right|}{\left|Y^{}\right|}.$$

#### (i) Fluid flow

We now consider the equations governing the fluid flow in $Y^{F}$ obtained at increasing orders of *ε*. At $O(\varepsilon^{-1})$ the equations are given by

3.28 $${\left(G_{}^{\left(0\right)}\right)}^{T}\nabla_{y}p^{\left(0\right)}=0$$

and

3.29 $$\nabla_{y}\cdot (G_{}^{\left(0\right)}v^{\left(0\right)})=0.$$

From (3.27), we observe that *p*^(0)^ is locally constant in $Y^{F}$, i.e. *p*^(0)^=*p*^(0)^(***x***,*t*). At O(1), we obtain the momentum equation

3.30 $$-{\left(G_{}^{\left(0\right)}\right)}^{T}(\nabla_{x}p^{\left(0\right)}+\nabla_{y}p^{\left(1\right)})+\mu \nabla_{y}\cdot D^{\left(0\right)}(v^{\left(0\right)})=J_{}^{\left(0\right)}f_{\varepsilon }^{F},$$

where ***D***^(*n*)^(⋅) denotes a modified rate of strain tensor defined by

3.31 $$D^{\left(n\right)}(\Upsilon ):=((\nabla_{y}\Upsilon )G_{}^{\left(n\right)}{\left(F_{}^{\left(n\right)}\right)}^{-T}+{\left(G_{}^{\left(n\right)}\right)}^{T}{\left(\nabla_{y}\Upsilon \right)}^{T}{\left(F_{}^{\left(n\right)}\right)}^{-T}).$$

Equations (3.29) and (3.28) now provide the so-called cell problem in $Y^{F}$ for the fluid.^3^ As such, we propose the following ansatz for the leading-order velocity and first-order pressure:

3.32 $$v^{\left(0\right)}=W_{1}\nabla_{x}p^{\left(0\right)}+W_{2}f_{\varepsilon }^{F}$$

and

3.33 $$p^{\left(1\right)}=\pi_{1}\cdot \nabla_{x}p^{\left(0\right)}+\pi_{2}\cdot f_{\varepsilon }^{F},$$

where ***W***_*i*_ and ***π***_*i*_ (*i*=1,2) are rank 2 tensors and vectors, respectively, that satisfy a pair of modified tensor Stokes problems, given by

3.34 $$\begin{array}{rlr} & -{\left(G_{}^{\left(0\right)}\right)}^{T}\nabla_{y}\pi_{1}+\mu \nabla_{y}\cdot D^{\left(0\right)}(W_{1})={\left(G_{}^{\left(0\right)}\right)}^{T} & \;\forall y\in Y^{F},\\ & \nabla_{y}\cdot (G_{}^{\left(0\right)}W_{1})=0 & \;\forall y\in Y^{F} \end{array}\}$$

and

3.35 $$\begin{array}{rlr} & -{\left(G_{}^{\left(0\right)}\right)}^{T}\nabla_{y}\pi_{2}+\mu \nabla_{y}\cdot D^{\left(0\right)}(W_{2})=J_{}^{\left(0\right)}I & \forall y\in Y^{F},\\ & \nabla_{y}\cdot (G_{}^{\left(0\right)}W_{2})=0 & \forall y\in Y^{F}, \end{array}\}$$

where ***W***_*i*_ and ***π***_*i*_ are ***y***-periodic, ***W***_*i*_=**0** on $Y^{\Gamma }$ and ***π***_*i*_ are mean-free on $Y^{F}$ for *i*=1,2.

The conservation of fluid mass at O(1) is given by

3.36 $$\nabla_{x}\cdot (G_{}^{\left(0\right)}v^{\left(0\right)})+\nabla_{y}\cdot (G_{}^{\left(1\right)}v^{\left(0\right)}+G_{}^{\left(0\right)}v^{\left(1\right)})=0.$$

To obtain the macroscopic equation governing the fluid, we substitute the ansätze given in (3.31) and (3.32) into the conservation of fluid mass equation given at O(1) and average over the reference cell. Application of the divergence theorem and ***y***-periodicity then yields

3.37 $$\nabla_{x}\cdot ({\left\langle G_{}^{\left(0\right)}v^{\left(0\right)}\right\rangle }_{Y^{F}})+{\left\langle (G_{}^{\left(1\right)}v^{\left(0\right)}+G_{}^{\left(0\right)}v^{\left(1\right)})\cdot n\right\rangle }_{Y^{\Gamma }}=0.$$

On a further application of the divergence theorem (this time on $Y^{S}$) and the substitution of the velocity interface condition given in (3.11), we obtain

3.38 $$\nabla_{x}\cdot ({\left\langle G_{}^{\left(0\right)}v^{\left(0\right)}\right\rangle }_{Y^{F}})+{\left\langle \nabla_{y}\cdot (G_{}^{\left(1\right)}g^{\left(0\right)}+G_{}^{\left(0\right)}g^{\left(1\right)})\right\rangle }_{Y^{S}}=0.$$

Finally, substituting in the ansatz for ***v***^(0)^ and the definitions of ***g***^(0)^ and ***g***^(1)^ we obtain

3.39 $$\nabla_{x}\cdot (K^{*}\nabla_{x}p^{\left(0\right)}+J^{*}f^{F})+g_{100}^{*}+g_{010}^{*}+g_{001}^{*}=0$$

in *Ω*, where

3.40 $$K^{*}:={\left\langle G_{}^{\left(0\right)}W_{1}\right\rangle }_{Y^{F}},\;J^{*}:={\left\langle G_{}^{\left(0\right)}W_{2}\right\rangle }_{Y^{F}},\;g_{ijk}^{*}:={\left\langle \nabla_{y}\cdot \left(G_{}^{\left(i\right)}F_{e}^{\left(j\right)}\frac{\partial {\overset{˚}{\chi }}^{\left(k\right)}}{\partial t}\right)\right\rangle }_{Y^{S}}.$$

Note that in (3.38) and (3.39) we are required to compute $F_{e}^{\left(1\right)}$ and $G_{}^{\left(1\right)}$, and that these quantities depend on $\nabla_{y}{\tilde{\chi }}^{\left(2\right)}$ and $\nabla_{y}{\overset{˚}{\chi }}^{\left(2\right)}$, and hence the system is not closed. Therefore, we are required to make appropriate closure assumptions. Here, we consider first order only correctors for $\tilde{\chi }$ and $\overset{˚}{\chi }$ in the definition of $F_{e}^{\left(1\right)}$ and $G_{}^{\left(1\right)}$, i.e. we replace these tensors in (3.38) and (3.39) with

3.41 $${\hat{F}}_{e}^{\left(1\right)}:={\hat{F}}_{e}^{\left(1\right)}({\tilde{\chi }}^{\left(0\right)},{\tilde{\chi }}^{\left(1\right)})\;\text{and}\;{\hat{G}}^{\left(1\right)}:={\hat{G}}^{\left(1\right)}({\tilde{\chi }}^{\left(0\right)},{\tilde{\chi }}^{\left(1\right)},{\overset{˚}{\chi }}^{\left(0\right)},{\overset{˚}{\chi }}^{\left(1\right)},c^{\left(0\right)},c^{\left(1\right)}).$$

We note that the validity of such closure assumptions will depend on the constitutive assumptions and parameter values employed in specific scientific and engineering applications. As such, we do not comment here on the domain of their applicability, but rather highlight this for consideration in future computational studies.

#### (ii) Elastic deformation

A corresponding macroscale description for the elasticity equations, parametrized by suitable microscale cell problems, is obtained by an almost identical process to that outlined in §3b(i) (see also [66] and the references cited therein). First, we define the Piola stress in the initial reference geometry by

3.42 $$T_{e}:=\sigma_{\varepsilon }^{S}G_{g}^{T}.$$

We now propose the expansion of the Piola stress of the form

3.43 $$T_{e}=\sum_{i=-1}^{\infty }\varepsilon^{i}T_{e}^{\left(i\right)},$$

which may be obtained by considering expansions for ***G***_g_ and $\partial \Psi (C_{e})/\partial C_{e}$ at increasing orders of *ε*. We note that this expansion runs from *i*=−1 as there is a spatial derivative implicit in the definition of $\sigma_{\varepsilon }^{S}$. At $O(\varepsilon^{-2})$ in (3.8), we obtain

3.44 $$-\nabla_{y}\cdot (T_{e}^{\left(-1\right)}({\tilde{\chi }}^{\left(0\right)},c^{\left(0\right)}))=0\;\forall y\in Y^{S}.$$

As $F_{g}^{\left(0\right)}$ and $G_{g}^{\left(0\right)}$ exhibit no *y*-dependence and we have restricted our choice of material such that the strain energy functional is strictly polyconvex, the resultant strong ellipticity of the operator [61] yields that the leading-order elastic deformation exhibits no microscale dependence (see [66–68] and associated literature discussing *Γ*-convergence [69–71]). From this observation, we deduce that $T_{e}^{\left(-1\right)}\equiv 0$ and that the proposed expansions for ***F***_e_, *J*_e_ and ***G***_e_ employed in [37] are also correct here.

Collecting terms at $O(\varepsilon^{-1})$, we obtain

3.45 $$-\nabla_{y}\cdot (T_{e}^{\left(0\right)}({\tilde{\chi }}^{\left(1\right)},{\tilde{\chi }}^{\left(0\right)},c^{\left(0\right)}))=0\;\forall y\in Y^{S}.$$

The coupling between the fluid and solid domains is given by the stress interface condition (3.13), from which we obtain

3.46 $$T_{e}^{\left(0\right)}({\tilde{\chi }}^{\left(1\right)},{\tilde{\chi }}^{\left(0\right)},c^{\left(0\right)})n=-p^{\left(0\right)}{\left(G_{}^{\left(0\right)}\right)}^{T}n\;\forall y\in Y^{\Gamma }.$$

Analogously to (3.31) and (3.32), we now propose the following ansatz for the first-order displacement:

3.47 $${\tilde{\chi }}^{\left(1\right)}=N(x,y):\nabla_{x}{\tilde{\chi }}^{\left(0\right)},$$

where N is a rank 3 tensor that satisfies the cell problem

3.48 $$-\nabla_{y}\cdot (T_{e}^{\left(0\right)}(N;{\tilde{\chi }}^{\left(0\right)},c^{\left(0\right)}))=0\;\forall y\in Y^{S},$$

subject to ***y***-periodicity and the interface condition

3.49 $$T_{e}^{\left(0\right)}(N;{\tilde{\chi }}^{\left(0\right)},c^{\left(0\right)})n=-p^{\left(0\right)}{\left(G_{}^{\left(0\right)}\right)}^{T}n\;\forall y\in Y^{\Gamma }.$$

Here, we consider (3.47) and (3.48) as the equations governing N, which is nonlinearly coupled to the macroscale quantities *p*^(0)^, $\nabla_{x}u^{\left(0\right)}$ and *c*^(0)^ via the implicit dependence of the stress on $F_{g}^{\left(0\right)}$ and $G_{g}^{\left(0\right)}$. In (3.47) and (3.48), all microscale derivatives $\nabla_{y}$ act on N only (both through divergence and the definition of ***F***_e_, and hence ***C***_e_). As such, we adopt the notation $T_{e}^{\left(0\right)}(N;\cdot )$ to clarify that this problem determines N, parametrized by the quantities appearing after the semi-colon that vary on the macroscale only.

At O(1), we then obtain

3.50 $$-\nabla_{y}\cdot T_{e}^{\left(1\right)}-\nabla_{x}\cdot (T_{e}^{\left(0\right)}({\tilde{\chi }}^{\left(1\right)},{\tilde{\chi }}^{\left(0\right)},c^{\left(0\right)}))=J_{g}^{\left(0\right)}f_{\varepsilon }^{S}.$$

Substituting in the ansatz (3.46) and taking the ***y***-average over $Y^{S}$ we obtain

3.51 $$-{\left\langle \nabla_{y}\cdot T_{e}^{\left(1\right)}\right\rangle }_{Y^{S}}-\nabla_{x}\cdot ({\left\langle T_{e}^{\left(0\right)}({\tilde{\chi }}^{\left(0\right)};N,c^{\left(0\right)})\right\rangle }_{Y^{S}})=J_{g}^{\left(0\right)}f_{\varepsilon }^{S},$$

where we now view $T_{e}^{\left(0\right)}$ as a function of ${\tilde{\chi }}^{\left(0\right)}$, parametrized by N and *c*^(0)^. Focusing on the first term in (3.50) and utilizing the divergence theorem, we obtain

3.52 $$\begin{array}{rl} -{\left\langle \nabla_{y}\cdot T_{e}^{\left(1\right)}\right\rangle }_{Y^{S}} & =-\frac{1}{\left|Y^{}\right|}\int_{Y^{\Gamma }}T_{e}^{\left(1\right)}n\;ds\\ & =\frac{1}{\left|Y^{}\right|}\int_{Y^{\Gamma }}{\left(\sigma_{\varepsilon }^{F}G_{}^{T}\right)}^{\left(1\right)}n\;ds\\ & ={\left\langle \nabla_{y}\cdot ({\left(\sigma_{\varepsilon }^{F}G_{}^{T}\right)}^{\left(1\right)})\right\rangle }_{Y^{F}}. \end{array}$$

In view of (3.52), and rewriting the fluid stress in terms of fluid pressure and velocity via (3.14) (and exploiting the ansätze (3.31) and (3.32)), we obtain the general homogenized equation governing solid mechanics given by

3.53 $$-\nabla_{x}\cdot {\left\langle T_{e}^{\left(0\right)}({\tilde{\chi }}^{\left(0\right)};N,c^{\left(0\right)})\right\rangle }_{Y^{S}}=J_{g}^{\left(0\right)}f_{\varepsilon }^{S}-(M_{1}^{*}-\alpha_{1}^{*}I)\nabla_{x}p^{\left(0\right)}-(M_{2}^{*}-\alpha_{2}^{*}I)f_{\varepsilon }^{F}+\beta^{*}p^{\left(0\right)}$$

in *Ω*, where

3.54 $$\begin{array}{rl} M_{i}^{*} & =\mu {\left\langle \nabla_{y}\cdot ((\nabla_{y}W_{i})G_{}^{\left(0\right)}{\left(F_{}^{\left(0\right)}\right)}^{-T}+{\left(G_{}^{\left(0\right)}\right)}^{T}(\nabla_{y}W_{i}){\left(F_{}^{\left(0\right)}\right)}^{-T})\right\rangle }_{Y^{F}}\\ \;\text{and}\;\alpha_{i}^{*} & ={\left\langle \nabla_{y}\cdot ({\left(G_{}^{\left(0\right)}\right)}^{T}\pi_{i})\right\rangle }_{Y^{F}},\;\beta^{*}={\left\langle \nabla_{y}\cdot {\left(G_{}^{\left(1\right)}\right)}^{T}\right\rangle }_{Y^{F}}. \end{array}\}$$

#### (iii) Solute transport

In this section, we consider the homogenization of the solute transport equations ((3.9) and (3.10)) in the periodic reference configuration. We proceed as in §3b(i),(ii) to obtain the equation governing the transport of solute at $O(\varepsilon^{-2})$

3.55 $$D\nabla_{y}\cdot (G_{}^{\left(0\right)}{\left(F_{}^{\left(0\right)}\right)}^{-T}\nabla_{y}c^{\left(0\right)})=0$$

throughout $Y^{}$, where we define

3.56 $$D=\left\{ \begin{array}{ll} D_{S} & \text{in }Y^{S}\\ D_{F} & \text{in }Y^{F}. \end{array}\right.$$

The interface condition at $O(\varepsilon^{-1})$ is given by

3.57 $$[[c^{\left(0\right)}]]=0\;\text{and}\;[[D\nabla_{y}c^{\left(0\right)}\cdot n]]=0.$$

Under the assumption of periodicity of $F_{e}^{\left(0\right)}$, $F_{g}^{\left(0\right)}$, $G_{e}^{\left(0\right)}$ and $G_{g}^{\left(0\right)}$, we conclude that *c*^(0)^ is independent of microscale variation, i.e. $c^{\left(0\right)}=c^{\left(0\right)}(x,t)$.

Collecting $O(\varepsilon^{-1})$ terms, we obtain

3.58 $$D\nabla_{y}\cdot (G_{}^{\left(0\right)}{\left(F_{}^{\left(0\right)}\right)}^{-T}(\nabla_{x}c^{\left(0\right)}+\nabla_{y}c^{\left(1\right)}))=0\;\forall y\in Y^{F}$$

and

3.59 $$D\nabla_{y}\cdot (G_{}^{\left(0\right)}{\left(F_{}^{\left(0\right)}\right)}^{-T}(\nabla_{x}c^{\left(0\right)}+\nabla_{y}c^{\left(1\right)}))=\nabla_{y}\cdot \left(J_{}^{\left(0\right)}{\left(F_{g}^{\left(0\right)}\right)}^{-1}\frac{\partial {\tilde{\chi }}^{\left(0\right)}}{\partial t}\right)c^{\left(0\right)}\;\forall y\in Y^{S},$$

subject to the interface conditions at O(1)

3.60 $$[[c^{\left(1\right)}]]=0\;\text{and}\;[[D(\nabla_{x}c^{\left(0\right)}+\nabla_{y}c^{\left(1\right)})\cdot n]]=0.$$

We propose the following ansatz for *c*^(1)^:

3.61 $$c^{\left(1\right)}=Q\cdot \nabla_{x}c^{\left(0\right)}.$$

Substituting (3.60) into (3.57)–(3.59), we obtain the cell problems for Q given by

3.62 $$D\nabla_{y}\cdot (G_{}^{\left(0\right)}{\left(F_{}^{\left(0\right)}\right)}^{-T}(I+{\left(\nabla_{y}Q\right)}^{T}))=0\;\forall y\in Y^{F}$$

and

3.63 $$D\nabla_{y}\cdot (G_{}^{\left(0\right)}{\left(F_{}^{\left(0\right)}\right)}^{-T}(I+{\left(\nabla_{y}Q\right)}^{T}))=f_{Q}\;\forall y\in Y^{S},$$

subject to the interface conditions

3.64 $$[[Q]]=0\;\text{and}\;[[D(I+{\left(\nabla_{y}Q\right)}^{T})n]]=0\;\forall y\in Y^{\Gamma }$$

and ***y***-periodicity over $Y^{}$, where the additional forcing in (3.62), $f_{Q}$, is given by

3.65 $$f_{Q}:=\left\{ \begin{array}{ll} \frac{\nabla_{y}\cdot (J_{}^{\left(0\right)}{\left(F_{g}^{\left(0\right)}\right)}^{-1}(\partial {\tilde{\chi }}^{\left(0\right)}/\partial t))c^{\left(0\right)}}{\sum_{i=1}^{d}(\partial c^{\left(0\right)}/\partial x_{i})}1 & \text{if }c^{\left(0\right)}\neq 0,\\ 0 & \text{if }c^{\left(0\right)}=0, \end{array}\right.$$

where 1 denotes a vector of length *d* whose entries are all 1. Collecting terms at O(1), substituting in the ansatz (3.60) and averaging over the reference cell $Y^{}$, we obtain the macroscale homogenized PDE governing the leading-order concentration of the solute *c*^(0)^, given by

3.66 $${\left\langle J_{}^{\left(0\right)}\right\rangle }_{Y^{}}\frac{\partial c^{\left(0\right)}}{\partial t}-V^{*}\cdot \nabla_{x}c^{\left(0\right)}=\nabla_{x}\cdot D^{*}\nabla_{x}c^{\left(0\right)}+R^{*}c^{\left(0\right)}$$

in *Ω*, where we define the effective advective velocity associated with growth by

3.67 $$V^{*}=(1+\phi ){\left(F_{g}^{\left(0\right)}\right)}^{-1}{\left\langle J_{}^{\left(0\right)}(I+{\left(\nabla_{y}Q\right)}^{T})\frac{\partial {\overset{˚}{\chi }}^{\left(0\right)}}{\partial t}\right\rangle }_{Y^{}},$$

the effective diffusivity by

3.68 $$D^{*}=D{\left\langle G_{}^{\left(0\right)}{\left(F_{}^{\left(0\right)}\right)}^{-T}(I+{\left(\nabla_{y}Q\right)}^{T})\right\rangle }_{Y^{}},$$

and the effective reaction by

3.69 $$R^{*}=(\phi -1)\left(R_{S}{\left\langle J_{}^{\left(0\right)}\right\rangle }_{Y^{S}}+{\left\langle \nabla_{y}\cdot {\left(J_{}F_{g}^{-1}\frac{\partial \overset{˚}{\chi }}{\partial t}\right)}^{\left(1\right)}\right\rangle }_{Y^{S}}\right).$$

We note that it may be possible to further rearrange (3.66)–(3.68) to precisely highlight which terms exhibit micro- and macroscale dependencies. We do not complete this process here, however, in order to maintain relatively compact expressions for the effective coefficients.

### (c) Summary of the model

To conclude this section, we briefly recall the equations that constitute the final coupled system. The cell equations for the fluid are given by (3.33) and (3.34), and the effective macroscale equation is given by (3.38). The cell equations for the solid are given by (3.47) and (3.48), and the effective macroscale equation is given by (3.52). Finally, the cell equations for the solute are given by (3.61)–(3.63), and the effective macroscale equation is given by (3.65).The flow, solid deformation and solute transport problems presented here are fully coupled. Growth and elastic deformation terms (***F***_e_, ***F***_g_, $\overset{˚}{\chi }$, etc.) appear explicitly in the fluid and solute transport equations. Further, growth, elastic deformation and fluid pressure terms occur in the solid mechanics equations.

## 4. Particular cases

In this section, we consider specific model reductions or parameter regimes so that we may compare the model derived in §3 with models considered elsewhere in the asymptotic homogenization literature. In particular, we consider comparison with the studies [35–38] and, therefore, restrict our attention to linearly elastic solids. In the light of our consideration of linear elasticity, we now introduce additional notation employed in this section. We define ${\overset{˚}{\sigma }}_{\varepsilon }^{S}$ constitutively by

4.1 $${\overset{˚}{\sigma }}_{\varepsilon }^{S}:=C:E_{\overset{˚}{x}}({\overset{˚}{u}}_{\varepsilon }),$$

where C denotes the fourth-order stiffness tensor for the material, ${\overset{˚}{u}}_{\varepsilon }$ denotes the displacement associated with the elastic deformation, and $E_{\overset{˚}{x}}(\psi )$ denotes the symmetric strain tensor

4.2 $$E_{\overset{˚}{x}}(\psi ):=\frac{1}{2}(\nabla_{\overset{˚}{x}}\psi +{\left(\nabla_{\overset{˚}{x}}\psi \right)}^{T}).$$

Given the displacement obtained via the solution of momentum equation (2.14), we define the linear elastic deformation by

4.3 $${\tilde{\chi }}_{\varepsilon }(\overset{˚}{x}):=\overset{˚}{x}+{\overset{˚}{u}}_{\varepsilon }\;\forall \overset{˚}{x}\in {\overset{˚}{\Omega }}_{\varepsilon }^{S}.$$

### (a) No growth

We first consider the case for which there is no growth, while explicitly retaining the (O(ε)) coordinate transformations corresponding to the elastic deformation. This asymptotic regime corresponds to physical situations in which the linear Biot model is applicable. As such, we refer the reader to [5] for an in-depth discussion regarding the applicability of this model to geomechanics. We note that under this assumption $F_{g}=G_{g}=I$ and *J*_g_=1; as such, we further observe that the quantities associated with the combined transformation become $F_{}=F_{e},G_{}=G_{e}$ and $J_{}=J_{e}$. Finally, the interface condition coupling the fluid and solid velocities is given by

4.4 $$v_{\varepsilon }=\frac{\partial u_{\varepsilon }}{\partial t}.$$

We observe that the system of equations governing flow and elasticity obtained under this regime in the formulation described in §3 reduce identically to those presented in [37]. Moreover, as remarked upon in [37], if we then consider the case of infinitesimal pore-scale deformation we obtain the standard Biot model of poroelasticity [6,35].

### (b) Finite growth, infinitesimal pore-scale deformation

We now consider the case of finite growth and infinitesimal pore-scale deformation as described in [36]. A potential application for this asymptotic regime is vascularized tissue growth, in which the solid material (a mixture of cells, interstitial fluid and extracellular matrix) undergoes small deformations and the fluid (blood) flows slowly. In the light of the transport considered here, we may further consider drug transport through the tissue and resulting cell death and tissue remodelling. Poroelastic frameworks have been employed in modelling solid tumours [1] and to extract poroelastic model parameters in biological experiments [72]. Moreover, in [73] a poroelastic model for cytoplasm of living cells is developed through experiment. Under this assumption $F_{e}=G_{e}=I$ and $J_{e}=1$, but $F_{g}\neq I$, $G_{g}\neq I$ and $J_{g}\neq 1$; as such, we are able to simplify the elasticity cell equations. Instead of (3.46), we now propose the following ansatz for the first-order displacement:

4.5 $$u^{\left(1\right)}=N(x,y):\nabla_{x}u^{\left(0\right)}-p^{\left(0\right)}R(x,y),$$

where N and R are a rank 3 tensor and vector, respectively, that satisfy the cell problems

4.6 $$-\frac{1}{2}\nabla_{y}\cdot ([C:((I+\nabla_{y}N){\left(F_{g}^{\left(0\right)}\right)}^{-1}+{\left(F_{g}^{\left(0\right)}\right)}^{-T}{\left(I+\nabla_{y}N\right)}^{T})]{\left(G_{g}^{\left(0\right)}\right)}^{T})=0\;\forall y\in Y^{S},$$

with the interface condition

4.7 $$\frac{1}{2}[C:((I+\nabla_{y}N){\left(F_{g}^{\left(0\right)}\right)}^{-1}+{\left(F_{g}^{\left(0\right)}\right)}^{-T}{\left(I+\nabla_{y}N\right)}^{T})]{\left(G_{g}^{\left(0\right)}\right)}^{T}n=0\;\forall y\in Y^{\Gamma };$$

and

4.8 $$-\frac{1}{2}\nabla_{y}\cdot ([C:(\nabla_{y}R{\left(F_{g}^{\left(0\right)}\right)}^{-1}+{\left(F_{g}^{\left(0\right)}\right)}^{-T}{\left(\nabla_{y}R\right)}^{T})]{\left(G_{g}^{\left(0\right)}\right)}^{T})=0\;\forall y\in Y^{S},$$

with the interface condition

4.9 $$\frac{1}{2}[C:(\nabla_{y}R{\left(F_{g}^{\left(0\right)}\right)}^{-1}+{\left(F_{g}^{\left(0\right)}\right)}^{-T}{\left(\nabla_{y}R\right)}^{T})]{\left(G_{g}^{\left(0\right)}\right)}^{T}n={\left(G_{g}^{\left(0\right)}\right)}^{T}n\;\forall y\in Y^{\Gamma },$$

together with ***y***-periodicity on $Y^{S}$, where I is the rank 4 tensor whose components are given by ${\left(I\right)}_{ijkl}=\delta_{ik}\delta_{jl}$.

We highlight that the macroscale dependence of N and R arises through $c^{\left(0\right)}(x)$ only, and all other macroscale dependence has been eliminated (cf. the cell problem for N given in (3.47) and (3.48)). Following an equivalent process to that described in §3b(ii), whereby we substitute the ansatz (4.5) into (3.44) and (3.45) and compute spatial averages, we obtain the macroscale elasticity equation

4.10 $$\begin{array}{rl} & -\frac{1}{2}\nabla_{x}\cdot ([C:(N^{*}\nabla_{x}u^{\left(0\right)}{\left(F_{g}^{\left(0\right)}\right)}^{-1}+{\left(F_{g}^{\left(0\right)}\right)}^{-T}{\left(N^{*}\nabla_{x}u^{\left(0\right)}\right)}^{T})]{\left(G_{g}^{\left(0\right)}\right)}^{T})\\ & \;+\frac{1}{2}\nabla_{x}\cdot ([C:(R^{*}p^{\left(0\right)}{\left(F_{g}^{\left(0\right)}\right)}^{-1}+{\left(F_{g}^{\left(0\right)}\right)}^{-T}{\left(R^{*}p^{\left(0\right)}\right)}^{T})]{\left(G_{g}^{\left(0\right)}\right)}^{T})\\ & \;=J_{g}^{\left(0\right)}f_{\varepsilon }^{S}+(\alpha_{1}^{*}I-M_{1}^{*})\nabla_{x}p^{\left(0\right)}+(\alpha_{2}^{*}I-M_{2}^{*})f_{\varepsilon }^{F}+\beta^{*}p^{\left(0\right)}, \end{array}$$

where, for *i*=1,2,

4.11 $$\begin{array}{rl} M_{i}^{*} & =\mu {\left\langle \nabla_{y}\cdot ((\nabla_{y}W_{i})G_{g}^{\left(0\right)}{\left(F_{g}^{\left(0\right)}\right)}^{-T}+{\left(G_{g}^{\left(0\right)}\right)}^{T}(\nabla_{y}W_{i}){\left(F_{g}^{\left(0\right)}\right)}^{-T})\right\rangle }_{Y^{F}},\\ \alpha_{i}^{*} & ={\left\langle \nabla_{y}\cdot ({\left(G_{g}^{\left(0\right)}\right)}^{T}\pi_{i})\right\rangle }_{Y^{F}},\;\beta^{*}={\left\langle \nabla_{y}\cdot {\left(G_{g}^{\left(1\right)}\right)}^{T}\right\rangle }_{Y^{F}}\\ \;\text{and}\;N^{*} & =I+{\left\langle \nabla_{y}N\right\rangle }_{Y^{S}},\;R^{*}={\left\langle \nabla_{y}R\right\rangle }_{Y^{S}}. \end{array}\}$$

Additionally, we must now re-specify the interface condition coupling the fluid and solid velocities as

4.12 $$v_{\varepsilon }=\frac{\partial {\overset{˚}{\chi }}_{\varepsilon }}{\partial t}+\frac{\partial u_{\varepsilon }}{\partial t}.$$

The macroscale flow equation is then given by

4.13 $$\begin{array}{rl} & \underset{I}{\underbrace{\nabla_{x}\cdot (K^{*}\nabla_{x}p^{\left(0\right)}+J^{*}f_{\varepsilon }^{F})}}+\underset{II}{\underbrace{{\text{g}}_{1}^{*}\cdot \left(\frac{\partial {\overset{˚}{\chi }}^{\left(0\right)}}{\partial t}+\frac{\partial u^{\left(0\right)}}{\partial t}\right)+{\text{g}}_{0}^{*}\cdot \frac{\partial {\overset{˚}{\chi }}^{\left(1\right)}}{\partial t}}}\\ & \;+\underset{III}{\underbrace{{\left\langle \nabla_{y}\cdot \left(G_{g}^{\left(0\right)}\frac{\partial N}{\partial t}\right)\right\rangle }_{Y^{S}}:\nabla_{x}u^{\left(0\right)}+{\left\langle \nabla_{y}\cdot (G_{g}^{\left(0\right)}N)\right\rangle }_{Y^{S}}:\nabla_{x}\frac{\partial u^{\left(0\right)}}{\partial t}}}\\ & \;-\underset{IV}{\underbrace{{\left\langle \nabla_{y}\cdot \left(G_{g}^{\left(0\right)}\frac{\partial R}{\partial t}\right)\right\rangle }_{Y^{S}}p^{\left(0\right)}-{\left\langle \nabla_{y}\cdot (G_{g}^{\left(0\right)}R)\right\rangle }_{Y^{S}}\frac{\partial p^{\left(0\right)}}{\partial t}}}=0, \end{array}$$

where

4.14 $$K^{*}={\left\langle G_{g}^{\left(0\right)}W_{1}\right\rangle }_{Y^{F}},\;J^{*}={\left\langle G_{g}^{\left(0\right)}W_{2}\right\rangle }_{Y^{F}}\;\text{and}\;{\text{g}}_{i}^{*}={\left\langle \nabla_{y}\cdot (G_{g}^{\left(i\right)})\right\rangle }_{Y^{S}}.$$

In (4.13), terms are labelled *I*–*IV* to facilitate comparison with the recent work [36], which considered a poroelastic medium, growing via a microscale (finite) accretion process represented by the following interface condition:

4.15 $$\left(v-\frac{\partial u}{\partial t}\right)\cdot n_{\varepsilon }=\eta ,$$

for accretion rate *η*. Under the assumption of infinitesimale pore-scale deformation (and adapting the notation employed in [36] to reflect that employed in this work) the following system of equations governing the coupled system was obtained:

4.16 $$\begin{array}{rl} & \nabla_{x}\cdot {\left\langle (C(\nabla_{y}N)+C):\nabla_{x}u^{\left(0\right)}+C:(\nabla_{y}R)p^{\left(0\right)}\right\rangle }_{Y^{S}}-\phi \nabla_{x}p^{\left(0\right)}\\ & \;+{\left\langle ((C(\nabla_{y}N)+C):\nabla_{x}u^{\left(0\right)}+C:(\nabla_{y}R)p^{\left(0\right)})q\right\rangle }_{Y^{\Gamma }}=0, \end{array}$$

and

4.17 $$\begin{array}{rl} \underset{I}{\underbrace{\nabla_{x}\cdot {\left\langle v^{\left(0\right)}\right\rangle }_{Y^{F}}}} & =\underset{II}{\underbrace{-{\left\langle \eta^{\left(1\right)}\right\rangle }_{Y^{\Gamma }}}}+\underset{III}{\underbrace{{\left\langle \nabla_{y}\cdot \left(\frac{\partial N}{\partial t}\right)\right\rangle }_{Y^{S}}:\nabla_{x}u^{\left(0\right)}+{\left\langle \nabla_{y}\cdot N\right\rangle }_{Y^{S}}:\nabla_{x}\frac{\partial u^{\left(0\right)}}{\partial t}}}\\ & \;+\underset{IV}{\underbrace{{\left\langle \mathrm{Tr}\;\frac{\partial \nabla_{y}R}{\partial t}\right\rangle }_{Y^{S}}p^{\left(0\right)}+{\left\langle \mathrm{Tr}\;\nabla_{y}R\right\rangle }_{Y^{S}}\frac{\partial p^{\left(0\right)}}{\partial t}}}+{\left\langle v^{\left(0\right)}\cdot q\right\rangle }_{Y^{\Gamma }}, \end{array}$$

where (4.16) describes the macroscale mechanics (where ***q*** accounts for macroscale variation in the interface) and (4.17) describes the macroscale flow.

Comparison of (4.10) and (4.13) against (4.16) and (4.17) highlights that, under finite growth and infinitesimal deformation, our formulation is structurally similar to that obtained in [36]. Differences arise in the following ways:

(i) For generality, we retain momentum sources ($f_{\varepsilon }^{F}$, $f_{\varepsilon }^{S}$), which are neglected in [36].
(ii) As we model growth coupled to solute transport (a feature not considered in [36]), N and R have macroscale dependence arising from a constitutively specified dependence on *c*^(0)^(***x***,*t*). Moreover, our (volumentric) model for growth differs, and as such there are corresponding differences in the relevant terms.
(iii) In [36], the constitutive assumption on the solid stress is given by $\sigma_{\varepsilon }^{S}=C:\nabla_{x}u_{\varepsilon }$; this simplifies the equations for the effective solid stress as there are no transpose terms.
(iv) Under the assumption of infinitesimal deformation, all equations in [36] may be posed in ${\overset{˚}{\Omega }}_{\varepsilon }$ and there is no requirement to map to an initial periodic geometry in order to perform the asymptotic homogenization. Correspondingly, terms involving ***F***_g_, ***G***_g_, etc. are not required in [36].
(v) The formulation in [36] contains additional terms on the interface (involving ***q***), which results from the extension to non-macroscopically uniform media via the application of the Reynolds transport theorem.

### (c) Infinitesimal growth, no deformation

The final case we consider is that of infinitesimal growth and no elastic deformation. A reasonable biological application in which growth is small and elastic deformation is sufficiently small that we may neglect it when considering flow and transport is that of bone tissue engineering. For instance, in [74] a rigid scaffold is seeded with human bone cells and cultured for four weeks. The resultant growth reduces the porosity of the structure by approximately 2%. Under this assumption, the resulting macroscale flow equation is then given by

4.18 $$\nabla_{x}\cdot (K^{*}\nabla_{x}p^{\left(0\right)}+J^{*}f^{F})+{\left\langle \nabla_{\text{x}}\cdot \frac{\partial {\overset{˚}{\chi }}^{\left(1\right)}}{\partial t}\right\rangle }_{Y^{S}}=0,$$

where

4.19 $$K^{*}={\left\langle W_{1}\right\rangle }_{Y^{F}}\;\text{and}\;J^{*}={\left\langle W_{2}\right\rangle }_{Y^{F}},$$

and the macroscale solute transport equation is given by

4.20 $$\frac{\partial c^{\left(0\right)}}{\partial t}=\nabla_{x}\cdot D^{*}\nabla_{x}c^{\left(0\right)}-(1-\phi )R_{S}c^{\left(0\right)},$$

where

4.21 $$D^{*}={\left\langle D(I+{\left(\nabla_{y}Q\right)}^{T})\right\rangle }_{Y^{}}.$$

In the recent work [38], a system of effective equations governing growing porous media is derived under the assumptions set out above, again considering growth via surface accretion. The form of the flow equation in [38] is identical to (4.18). However, the solute transport equation differs due to the choice of time scale (time associated with macroscopic advection) adopted in [38].

## 5. Conclusion

In this article, we have performed a spatial homogenization of a coupled fluid–structure interaction and solute transport model applicable to the study of active poroelastic media. The effective description that we obtain is of wide relevance to problems in, for example, tissue engineering, geophysics and industry.

The multiple-scale techniques exploited in this work have been widely applied in the homogenization of flow and transport in porous media [35,53] and more recently to restricted models of growing media [36,38,39]. Here, we extend these ideas to a significantly more complex description of the underlying dynamics. By mapping the fluid–structure interaction and transport systems to a common frame, an approach also adopted in [37,41–43], we are able to derive an effective description on the macroscale. We then investigated the correspondence, under selected simplifying asymptotic limits, between our resulting macroscopic description and other recent models in the literature.

There are a number of natural extensions to the work described in this article. The most significant of these is the investigation of this model via numerical experiments. At present, there exist relatively few three-dimensional computational examples of typical cell problems arising from asymptotic homogenization (e.g. [40,75–78]). The cell problems associated with this work are considerably more complex; furthermore, the coupling between the macroscale variables and the cell problems represent a significant challenge. However, given that the macroscopic variables are constant on the cell, we may view these as high-dimensional parameteric PDEs, and, as such, it may be possible to reduce computational load through the utilization of empirical interpolation methods [79]. These methods employ greedy algorithms to determine which parameters contain key information on lower-dimensional structures in the parameter space. Alternatively, model order reduction methods such as proper orthogonal decomposition (POD) and dynamic mode decomposition (DMD) [80–82] may be employed to obtain a reduced model, whereby snapshots of solutions are taken across the parameter space, and singular value decompositions are employed to extract lower-dimensional structures.

The challenge of parametrizing three-dimensional geometries is a highly active area of research at the frontier of multiscale analysis in porous media. It is currently unclear how to correctly extract parametrizations of deformation and growth from computational data. One possible route forward may be to consider decoupled processes (such as flow, mechanics, transport, etc.), considering each phenomenologically, whereby a parametrization may be ascertained from physical assumptions. These ideas have been employed in the case of flow in complicated microstructures that have evolved via decoupled processes [83–85]. In [83,84], the authors employ a reduced basis to reduce the order of their model under the assumption of a physical parametrization of their geometry. In [85], the authors consider a geometry deformed via fluid–structure interaction to decouple the flow and mechanical processes, and subsequently employ homogenization and corrector techniques to construct a more efficient computational scheme.

Upon the development of suitable computational schemes, we emphasize the importance of comparison of our effective description with the underlying true description given on the microscale. Perhaps the most important piece of future work is, however, the full parametrization of this model against experimental data across a range of application areas, in order to validate its use.

In addition to the future work associated with the numerical simulations, we also highlight several theoretical extensions. The first of these is the inclusion of thermoelasticity, whereby the constitutive statement on the solid stress exhibits dependence on temperature (e.g. [60]). While the model will be structurally similar to that studied in this work, there are differences in the analysis and, as such, we defer its consideration. The second is the inclusion of multiple phases in the solid material, i.e. we model the solid as a multiphase mixture. This approach lends itself to a more precise description of biological tissues (modelling various cellular materials and extracellular matrix as separate phases). Moreover, such a formulation allows for a more natural description of conservation of mass between solid and fluid phases that would provide more direct applicability to swelling/growth applications of interest here. We remark, however, that in this case the strain energy functional may not exhibit the correct ellipticity properties to permit a fully nonlinear analysis. The third is the coupling of this model to those presented in [86,87] to model drug transport in vascularized tumours through the adoption of a double porous medium approach. The final theoretical extension we propose is the inclusion of solid stress in the constitutive statement of the growth law. The potential difficulty associated with this formulation arises while demonstrating that the leading-order elastic deformation is macroscale.

## Footnotes

1 For notational convenience, we refer to the mass/volumetric changes of the solid material as growth for the remainder of this article. While this is natural in the context of biological materials, it is less so in physical and chemical applications. However, for the sake of brevity we employ this single term.

2 We mention these time scales here only to maintain consistency with the discussion in [54].

3 Where we define a cell problem as a system defined on the periodic cell $Y^{}$, which allows the determination of the microscopic variation of non-leading-order quantities, so that we may specify effective macroscopic coefficients.

## Appendix A. Nomenclature

Given the volume of nomenclature employed in this work, table 1 contains a brief description of the key notation adopted. We note that table 1 is not exhaustive; however, it should be clear how to compose elements with one another to generate all notation employed here, for instance in the definition of the solid portion of the grown domain ${\overset{˚}{\Omega }}_{\varepsilon }^{S}$, etc.
